# Phase I Study in Healthy Women of a Novel Antimycotic Vaginal Ovule Combining Econazole and Benzydamine

**DOI:** 10.1155/2020/7201840

**Published:** 2020-05-02

**Authors:** A. F. D. Di Stefano, M. M. Radicioni, A. Vaccani, G. Caccia, F. Focanti, E. Salvatori, F. Pelacchi, R. Picollo, M. T. Rosignoli, S. Olivieri, G. Bovi, A. Comandini

**Affiliations:** ^1^CROSS Research S.A., Via F. A. Giorgioli, 14, CH-6864 Arzo, Switzerland; ^2^Service for Gynaecology and Obstetrics, Ospedale Regionale di Mendrisio, Mendrisio, Switzerland; ^3^Angelini S.p.A., S. Palomba, Rome, Italy

## Abstract

**Purpose:**

A novel fixed-dose combination of 150 mg of econazole with 6 mg of benzydamine formulated in vaginal ovules was investigated in a randomised, double-blind, four-parallel group, tolerability, and pharmacokinetic Phase I study in healthy women.

**Methods:**

The fixed-dose combination was compared to econazole and benzydamine single-drug formulations and with placebo after daily applications for 3 consecutive days. Safety and tolerability were evaluated recording the adverse drug reactions, local and general tolerability scores, clinical laboratory assays, and vital signs. Econazole, benzydamine, and its metabolite benzydamine N-oxide pharmacokinetics were investigated after single and multiple applications.

**Results:**

Local reactions were generally absent. Pruritus and pain at the application site were infrequently reported. According to the subjects' evaluations, the overall tolerability of the ovules was rated as excellent or good. No significant effect of any treatment on laboratory parameters, vital signs, body weight, vaginal pH, or ECG was observed. Very low econazole, benzydamine, and benzydamine-N-oxide concentrations were measured in plasma, though quantifiable in almost all samples.

**Conclusion:**

The tested fixed-dose combination showed a good safety profile consistently with the known tolerability of both active substances. In addition, the confirmed low bioavailability of the drugs excludes the possibility of any accumulation effects and limits the risk of undesired systemic effects. This trial is registered at ClinicalTrials.gov with the identifier NCT02720783 last updated on 07 February 2017.

## 1. Introduction

Vulvovaginal mycotic infections are typically caused by *Candida albicans* [1] or non-*albicans* species [2–7] and are generally defined as vulvovaginal candidiasis. With respect to the epidemiology of vaginal infections due to various *Candida* species, Richter et al. found that the presence of *C. albicans* was 70.8%, followed by *C. glabrata* (18.9%) and *C. parapsilosis* (5%) in the USA [1]. A survey conducted by Corsello et al. confirmed that the disease prevalence is similar in Italy, with *C. albicans* at 77.1%, followed by *C. glabrata* (14.6%) and *C. krusei* (4%) [5].

In particular, *C. albicans* vulvovaginitis is a very frequent ailment [8–10], as it is estimated that approximately three quarters of all women will suffer from at least one episode of vulvovaginal candidiasis during their lifetime and that about 5% will experience the so-called *recurrent vulvovaginal candidiasis* (4 or more episodes over one-year periods) [11–13].

The novel vaginal ovule investigated in this study combines econazole, an approved drug effective against vulvovaginal candidiasis, with benzydamine, a drug endowed with local anaesthetic and anti-inflammatory properties.

Econazole is an antimycotic imidazole derivative synthesized in 1969 and introduced into the market in the seventies all over the world. Econazole has a wide spectrum of activity against many common dermatophytes, *Candida* species, yeasts, actinomycetes, moulds, and other filamentous fungi (*Aspergillus* and *Rhizopus spp*) as well as some Gram-positive bacteria [14–18]. Econazole primarily affects the cell membrane of mycetes preventing the synthesis of ergosterol [19–22]. Exposure of fungi to econazole causes depletion of ergosterol, accumulation of 14*α*-methylated sterols [19–21], and, finally, inhibition of their growth [21]. In the literature, the pharmacokinetics of econazole was almost exclusively studied after dermal or intravaginal application with a rather limited overall amount of data. The enteral use of econazole is restricted by its low systemic bioavailability due to extensive plasma protein binding [23]. Peak plasma econazole levels after vaginal application of marketed 150 mg vaginal ovules were 65 ng/mL [24].

Benzydamine was developed at the Angelini laboratories in 1967. The active substance is endowed with anti-inflammatory, local analgesic, and anaesthetic properties and is nowadays available in different formulations in many countries worldwide. The pharmacological activity of benzydamine occurs through a block of cation channels, responsible for stimulus transmission along nervous fibres, and through inhibition of proinflammatory cytokine synthesis. The efficacy of a 0.1% benzydamine vaginal solution in the treatment of vulvovaginitis was proven in several clinical studies [25–28].

Literature studies of vaginal benzydamine douches demonstrated their safety and a low rate of systemic absorption [29]. Following a single intravaginal application, only a minimal percentage of the drug was detectable in plasma, compared to vaginal mucosa [29–32].

A 3-day course of therapy with econazole 150 mg vaginal ovules is the approved dose regimen for the treatment of uncomplicated vulvovaginal candidiasis. With respect to benzydamine dose choice, 6 mg demonstrated a clear anaesthetic effect and is a well-tolerated dose after vaginal application. The dose of 6 mg of benzydamine was tested as a vaginal cream containing 0.12% benzydamine and 1% econazole nitrate in *in vitro* and *in vivo* preclinical tests and demonstrated to be the highest tolerated dose. Moreover, the 0.12% concentration retains full anaesthetic activity thus providing the combination product with an early relieving effect on vulvovaginal itching, burning, and irritation [33]. In addition, a vaginal solution containing a similar benzydamine concentration (0.1%) is currently marketed for the treatment of vulvovaginitis of any origin and nature [34].

The combination of econazole with benzydamine is aimed at maintaining the same efficacy in eradicating yeasts as other available treatments, due to the presence of the antimycotic agent, while providing a faster relief of the symptomatology thanks to benzydamine.

The present exploratory Phase I study allowed collecting preliminary data about the local and general tolerability of the novel fixed-dose combination, applied for 3 days by healthy women. In addition, the pharmacokinetic profile of both econazole and benzydamine was investigated in order to confirm their scarce systemic absorption when applied intravaginally.

## 2. Methods

### 2.1. Study Design

The present study was a multiple-dose, randomised, double-blind, four-parallel group, tolerability, and pharmacokinetic Phase I study in healthy women. The primary objective was the investigation of local tolerability of the test fixed-dose combination ovules in comparison with 150 mg econazole and 6 mg benzydamine single-drug formulations and with placebo after daily applications for 3 consecutive days. During the study, adverse drug reactions, clinical laboratory tests, and vital signs were evaluated to assess the safety and tolerability of the new fixed-dose combination.

The pharmacokinetic profile of econazole, benzydamine, and its main metabolite N-oxide benzydamine was investigated after single and multiple applications of the test and of the econazole and benzydamine single-drug formulations. Each subject underwent a screening visit, then, if eligible, was confined from the evening of Day 1 to the morning of Day 5, when the completers underwent the final visit and were definitely discharged.

#### 2.1.1. Study Population and Criteria for Inclusion

The study was performed at the Phase I Unit of CROSS Research S.A., Arzo, Switzerland.

Healthy women were included into the trial according to the following main inclusion criteria: (i) age of 18 to 55 y; (ii) body mass index inside the range 18.5-30 kg/m^2^; (iii) good health based on medical history, physical examination, 12-lead electrocardiogram (ECG), and routine haematology and blood chemistry tests; (iv) use of highly effective contraceptive methods for at least 2 months prior to the study start [35]; and (v) willingness to provide written informed consent.

Main exclusion criteria were the standard criteria for bioavailability estimation of drugs and, in particular, for the evaluation of the tolerability of vaginal ovules: (i) intake of any medication; (ii) history of drug, caffeine (>5 cups coffee/tea/day), or tobacco (≥10 cigarettes/day) abuse; (iii) history of alcohol consumption in excess of one drink per day, as defined by the USDA dietary guidelines [36]; (iv) genitourinary diseases; (v) systemic and/or local infections; (vi) clinically significant conditions affecting the vagina; and (vii) positive Pap test.

The study was descriptive and noncomparative. Therefore, the sample size was not derived from a statistical power calculation. A total of 60 subjects were planned to be included in the study in order to have 48 completers (12 in each treatment arm) for the analysis.

#### 2.1.2. Investigational Treatments and Dose Regimen

The randomised subjects applied once a day for 3 consecutive days 1 of the 4 following treatments manufactured by Angelini: (i) the test (T) fixed-dose combination econazole nitrate 150 mg/benzydamine hydrochloride 6 mg vaginal ovules, (ii) the reference 1 (R1) vaginal ovules containing only econazole nitrate 150 mg, (iii) the reference 2 (R2) vaginal ovules containing only benzydamine hydrochloride 6 mg, or (iv) placebo (P), i.e., vaginal ovules of hard fat (Witepsol H15) not containing any active substance (R1, R2, and P were manufactured by Angelini only for this clinical trial).

The volunteers were assigned to 1 of 4 treatments according to the randomisation list and the parallel group design.

The ovules were inserted intravaginally, o.d. (once daily), in the evening for 3 consecutive days, for a total of 3 applications from Days 1 to 3 of the study. For each application, one ovule (2.7 g) was inserted deep into the vagina. Time of application was 20 : 30 ± 1.5 h.

Each application was performed by the subjects during their confinement under the supervision of the investigator.

The first application was performed after at least one week from screening. Fertile women applied their treatment away from the menstrual cycle.

Both the investigator and the subjects were unaware of the allocated treatment. Adequate procedures were put in force to maintain the blind conditions and to avoid any data disclosure before the clinical database lock. The determination of econazole, benzydamine, and its metabolite in plasma was performed in blind conditions. Pharmacokinetic evaluations were performed only after the clinical database lock.

### 2.2. Ethical Procedures

The study was approved by the independent ethics committee of Canton Ticino on 14 December 2015, Ref. no. 2999. The Swiss Federal Health Authorities (Swissmedic) authorised the study on 4 February 2016 and assigned the reference number 2016DR1024. The study was conducted in compliance with the Swiss ordinance on clinical trials in human research (OSRUm) and in accordance with the Declaration of Helsinki and the general principles of ICH Guidelines for GCP. Subjects did not undergo any study procedure before signing the written informed consent form. The first subject was enrolled in March 2016, and the last subject completed the trial in October 2016.

### 2.3. Safety Variables

Blood pressure and heart rate were measured on treatment days at predose and at 12 and 24 h postdose and upon subjects' discharge. ECGs and routine clinical laboratory parameters including haematology, blood chemistry, and urine analysis were measured at screening and at final visit.

Vaginal pH was measured using pH colour-fixed indicator strips at screening, before the daily ovule application, and at the final visit.

A gynaecological visit was performed by the study gynaecologist before the start of the treatment and at the end of the study. The screening gynaecological visit included a Pap test and a vaginal sample to analyse the following microbiota: *C. albicans*, *C. spp*, *Gardnerella vaginalis*, beta-haemolytic streptococci, *Gonococcus*, *Actinomyces*, *Ureaplasma*/*Mycoplasma*, and *Chlamydia trachomatis*. If the results of the microbiological analyses concerning the above-mentioned strains were positive at the screening visit, the subject was enrolled only if asymptomatic and without any clinical evidence of vaginal infection according to the results of the gynaecological visit, Pap test, and vaginal pH measurement. In order to evaluate possible specific genitourinary symptoms before the study treatment, the subjects were questioned upon confinement by the investigator who asked specific and standardised questions about well-being; genital/vulvovaginal symptoms, such as itching, burning sensation, dryness, pain, vaginal discharge, and foul odour; and urinary symptoms, such as urgent, frequent, difficult, painful, or burning urination and pain on either back side.

During the treatment, all the application site signs or symptoms were reported by the subjects. In addition, the subjects evaluated the local tolerability immediately before and after each daily application, by giving a score to application site pruritus, burning sensation, pain, stinging, and dryness using a 0-3 scoring system for their severity.

At the end of the study, the gynaecologist and the subjects evaluated the overall local tolerability of the treatment using a 5-point scale rating it from score 1, i.e., excellent (no skin reaction), to 5, i.e., bad (serious skin reaction).

The adverse events were recorded throughout the study. Particular attention was given to events and reactions occurring immediately after the treatment application.

### 2.4. Comfort of Use Evaluation

In order to evaluate the comfort of use, after the end of the treatment, the investigator asked the subjects a list of questions: (i) Does the product easily dissolve? (ii) Does the product flow out of the vagina? (iii) Does the product grease? (iv) Is the product sticky? (v) Does the product maculate? (vi) How does the product smell?

### 2.5. Sample Collection and Analytic and Pharmacokinetic Variables

Plasma concentrations of econazole, benzydamine, and its major metabolite benzydamine N-oxide were measured at predose (0) and 1, 2, 3, 4, 6, 8, 12, and 24 h postdose, both on Days 1-2 after the first dose and on Days 3-4 after the last dose.

Predose samples (Days 1 and 3) and 24 h postdose samples after the first dose (Day 2) were collected within 30 min before the subsequent treatment application.

Econazole, benzydamine, and benzydamine N-oxide AUC_0‐*t*_ was calculated, where feasible, from the application time to the time of the last measurable concentration.

The pharmacokinetic analysis was performed considering the time at predose as zero and the times after dosing as the actual sampling times for each subject and treatment, using a noncompartmental method (linear trapezoidal and linear interpolation method) and with the validated software Phoenix-WinNonlin (Pharsight-Certara™) version 6.4.

Blood samples were collected using an indwelling catheter with switch valve and transferred into prechilled labelled tubes containing potassium EDTA (K2EDTA) as the anticoagulant. The samples were stored on ice no longer than 20 min and then centrifuged at 4°C for 10 min at 2500 × g to obtain plasma. Each plasma sample was divided into aliquots in polypropylene tubes and stored frozen within 45 min from the time of centrifugation and kept at −80 ± 15°C until shipment to the bioanalytical laboratory, Pharma Medica Research Inc., Canada, where the concentration of econazole (free base), benzydamine (free base), and benzydamine N-oxide (free base) in plasma was determined by a fully validated HPLC-MS/MS method involving protein precipitation out of 0.100 mL of plasma with a lower quantification limit of 0.05 ng/mL for benzydamine and benzydamine N-oxide and 2.5 ng/mL for econazole.

## 3. Results

### 3.1. Disposition of Subjects

Forty-nine (49) women, aged 21 to 55 y, were enrolled. The study was prematurely terminated before completing the inclusion of 60 women, as planned, due to slow enrolment. Baseline demographic data are summarised in Table 1.

All 49 enrolled subjects received the planned treatment and completed the study as per protocol. All the 49 subjects were included in the statistical analyses of safety and pharmacokinetic data and in the analysis of comfort of use.

### 3.2. Econazole Plasma Concentration after Single and Multiple Doses

**Figure 1:**
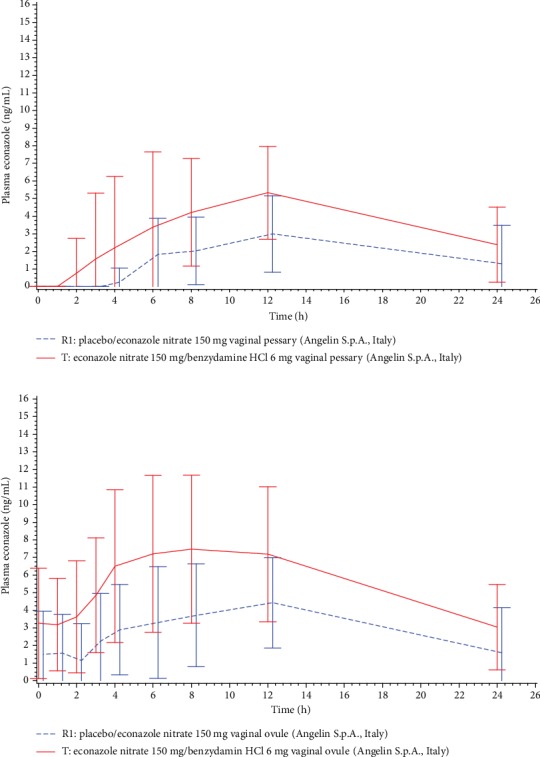
Mean (±SD) econazole concentration (ng/mL) vs. time profiles on Day 1 of treatment with T (*N* = 10) and R1 (*N* = 6) (top) and on Day 3 of treatment with T (*N* = 12) and R1 (*N* = 12) (bottom). Linear scale.

**Figure 2:**
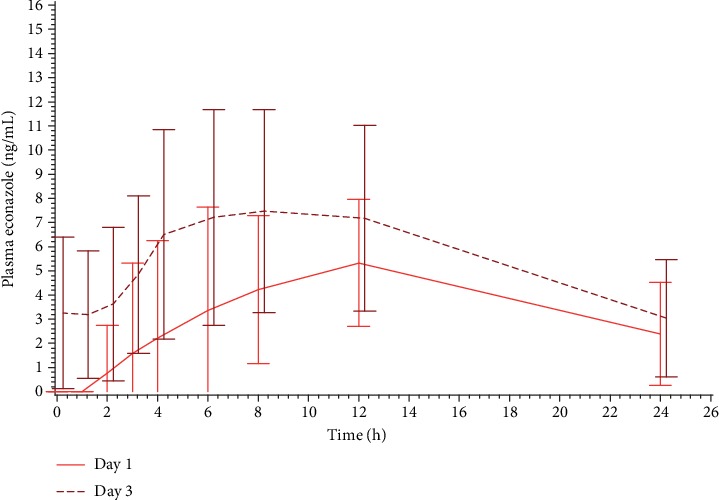
Mean (±SD) econazole concentration (ng/mL) vs. time profiles on Days 1 (*N* = 10) and 3 (*N* = 12) of treatment with T. Linear scale.

Econazole plasma levels after the first and the last application of T and R1 are shown in Figure 1 in a linear scale, while the comparison of econazole plasma levels on Day 1 versus Day 3 after the first and the last application of T is shown in Figure 2. AUC_0‐*t*_ of plasma econazole on Days 1 and 3 of the treatment is presented in Table 2.

The excluded subjects did not have any quantifiable concentration or had only one or 2 quantifiable concentration values postdose.

After the 3^rd^ and last application of vaginal ovules, all the subjects applying R1 and 12 subjects applying T showed at least one quantifiable postdose concentration value. Most subjects applying T (9) and 4 subjects applying R1 still had quantifiable concentrations 24 h postdose, while for the remaining subjects, the analyte concentrations decreased under the limit of quantification after 12 h postdose.

Econazole concentrations were plotted vs. time for 12 subjects in each treatment group. The excluded subject, who applied T, did not have any quantifiable concentration value postdose.

### 3.3. Benzydamine Plasma Concentration after Single and Multiple Doses

**Figure 3:**
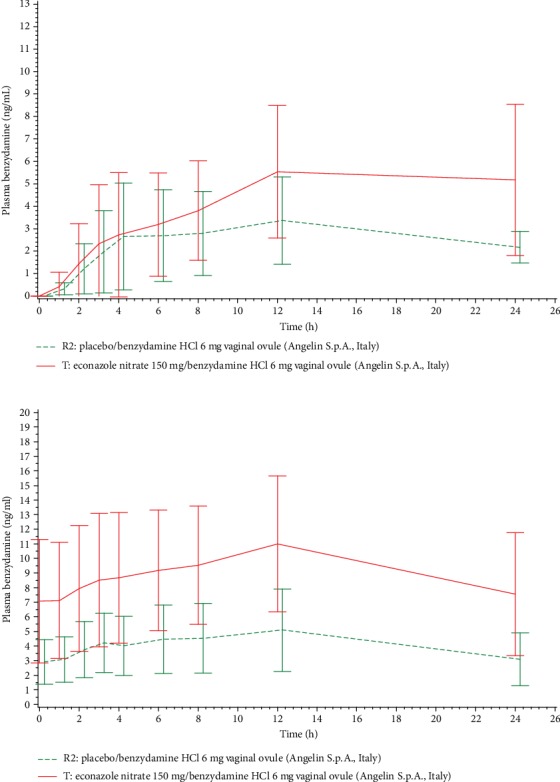
Mean (±SD) benzydamine concentration (ng/mL) vs. time profiles on Day 1 (top) and on Day 3 of treatment with T (*N* = 13) and R2 (*N* = 12) (bottom). Linear scale.

**Figure 4:**
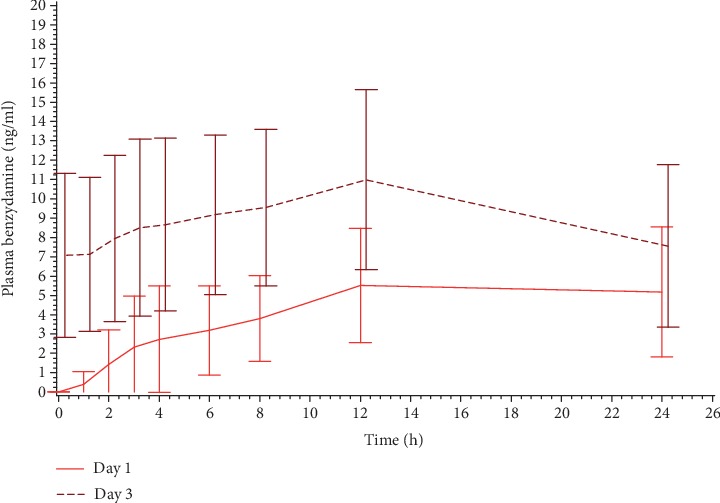
Mean (±SD) benzydamine concentration (ng/mL) vs. time profiles on Days 1 and 3 of treatment with T (*N* = 13). Linear scale.

Benzydamine plasma levels after the first and the last application of T and R2 are shown in Figure 3 in a linear scale, while the comparison of benzydamine plasma levels between Day 1 and Day 3 after use of T is shown in Figure 4. Benzydamine N-oxide plasma levels had a behaviour very similar to those of benzydamine (data not shown). AUC_0‐*t*_ of plasma benzydamine and benzydamine N-oxide on Days 1 and 3 of the treatment is presented in Table 2.

On treatment Day 1, all the subjects applying T and R2 showed the first quantifiable benzydamine concentration between 1 and 2 h postdose.

All the subjects showed quantifiable benzydamine concentrations up to 24 h postdose.

On treatment Day 3, all the subjects showed quantifiable benzydamine concentrations from the predose measurement up to 24 h postdose.

### 3.4. Comfort of Use

The comfort of use of the fixed-dose combination was very similar to that of the other study treatments.

In particular, T easily dissolved and did not stick for all the subjects. All 13 subjects using T reported that the ovules did not smell. Almost the totality of the subjects (92.3%) responded that the product did not flow. About half of the subjects (53.8%) found that the product greased. The product was found not to maculate by three-quarters of the subjects (76.9%). Similar results were reported in the other 3 treatment groups.

### 3.5. Local Tolerability and Adverse Events and Reactions

The reported adverse events are summarised by system organ class and preferred term in Table 3.

Local reactions were absent for most subjects during the whole duration of the study. Remarkably, no subject reported either burning sensation or pain or dryness at the application site for the whole duration of the study.

The most frequently reported disorder was an otherwise unspecified application site reaction which occurred to 4 subjects (33.3%) applying R1. Notably, 2 of these had concurrent positive microbiological results: *β*-haemolytic streptococci and *G. vaginalis*, respectively. The second most frequent reaction was pruritus at the application site reported by 2 out of 13 subjects (15.4%) after the 1^st^ and the 2^nd^ application of T, respectively, and by another subject (8.3%: one out of 12 subjects) after the 2^nd^ application of R1. No disorder at the application site was reported in either the R2 or the placebo group.

All these local reactions were defined as related to the study treatment by the investigator and altogether were 3 reactions after application of T (3 subjects, 23.1%) and 5 after R1 (4 subjects, 33.3%).

No specific therapeutic countermeasures were taken against the occurred reactions, and all the events resolved spontaneously.

All other unrelated adverse events which included headache, dizziness, nausea, and some episodes of positive microbiological results which were reported after the end of the treatment at the final laboratory examination occurred at a frequency lower than 10%.

Indeed, the individual clinical laboratory assays in haematology, blood chemistry, urine analysis, microbiological examination, and molecular biology at screening were normal or abnormal but devoid of clinical significance for all the enrolled subjects. At the final visit, the found abnormalities were judged nonclinically significant by the investigator with the exception of positive results of microbiological assays found in 5 subjects (Table 3). In detail, *β*-haemolytic streptococci and *G. vaginalis* in the R1 group, *G. vaginalis* and *C. albicans* in the R2 group, and *Ch. trachomatis* among the placebo recipients were reported as adverse events unrelated to the study treatments.

No notable change in any other laboratory parameter was observed between the screening and the final laboratory analysis.

All the reported microbiological infections resolved after application of specific therapeutic countermeasures with the exception of one infection of *G. vaginalis* which resolved spontaneously. Notably, no clinically significant microbiological abnormality was found in the subjects treated with the fixed-dose combination. The investigator judged the found vaginal infections as unrelated to the study treatment and mild in severity.

### 3.6. Other Safety Measures

Overall tolerability was scored by both the gynaecologist and the subjects as excellent or good for all subjects in all 4 treatment groups with the exception of one subject applying R1. Both this subject and the gynaecologist scored the overall local tolerability of the treatment as moderate, and the disorder was reported as an adverse event (verbatim: application site reaction) moderate in severity with unknown duration, which resolved spontaneously in a few days. No scores for bad or poor tolerability were recorded.

No relevant effects of the study treatments on blood pressure, heart rate, body weight, vaginal pH, or ECG results were observed. In particular, vaginal pH remained constantly around 4.2-4-5 during the whole duration of the study.

## 4. Discussion

The novel fixed-dose combination ovules, containing 150 mg of econazole and 6 mg of benzydamine, proved to be safe and very well tolerated by healthy women after 3 days of treatment. Local reactions were absent for most subjects during the whole duration of the study. Indeed, episodes of pruritus and of pain at the application site were reported infrequently (≤15% of the subjects). With econazole alone, application site reactions occurred more frequently (33%). Furthermore, in the opinion of the subjects and of the gynaecologist, the overall tolerability of the fixed-dose combination ovules was generally excellent or good. With respect to the clinical laboratory tests, no significant effect was observed following 3 days of treatment. In particular, the vaginal microbiology assays at the end of the study treatment were mostly normal, since abnormal microbiological findings were few (8.3%) in each treatment group. No relevant effects on blood pressure, heart rate, body weight, vaginal pH, or ECG results were observed.

With respect to the subjective evaluation performed by the women who applied the fixed-dose combination ovules, it was reported that the new formulation easily dissolved, did not stick, and did not flow out. The women reported that the product did not smell, and about half of them reported that the formulation did not grease. The evaluations of the comfort of use of the other 3 tested products were generally similar.

Taking into account the above results, a good tolerability profile was found for all the tested formulations, with a slightly higher frequency of local reactions in subjects applying the econazole single-drug product. However, no relevant safety concerns arose during the treatment with either study formulation. Indeed, neither severe adverse events nor serious adverse events occurred during the study. No subject discontinued the study due to safety reasons.

Quantifiable plasma levels of econazole were very limited after the 1^st^ application of the fixed-dose combination ovules and were slightly higher after the 3^rd^ and last application, thus confirming that the systemic absorption of econazole is very low, as shown by the previous literature [14, 37–39]. Econazole plasma levels were slightly higher after the fixed-dose combination than after the econazole single-drug product. After 3 consecutive applications, accumulation of plasma econazole was considered negligible.

The concentrations of both benzydamine and its metabolite benzydamine-N-oxide were very low, though quantifiable in all the samples after the 3^rd^ and last application and 1 or 2 h after the 1^st^ application. Plasma levels were slightly higher after the application of the fixed-dose combination than after the benzydamine single-drug product. As for econazole, also benzydamine plasma concentrations showed a negligible accumulation. The profile of the benzydamine metabolite, benzydamine N-oxide, was similar to that of the parent compound.

The higher absorption of both active ingredients from the fixed-dose combination ovules, in terms of AUC_0‐*t*_ and peak values, as compared to the econazole and benzydamine single-drug products, could be explained by the results of a dedicated dissolution test aimed at clarifying the higher release from the combination ovules.

It is important to underline that benzydamine is solubilised in water during the manufacturing process, and once added to the melted fatty mass, an emulsion forms to which insoluble econazole is added as a suspension. Probably, the presence of econazole salts near the interface alters the emulsion facilitating a faster release of both active ingredients. The differences observed in terms of release are more evident for benzydamine because, in the combination, it is promptly available in solution, differently from econazole which is present in suspension.

According to the above-exposed considerations, higher AUC_0‐*t*_ and peak values of econazole and benzydamine observed with the combination in comparison with values obtained with the single-drug products can be explained by the interaction between econazole salts and emulsion-containing benzydamine: once the ovule comes into contact with the vaginal fluid, both benzydamine and econazole present at the interface are released faster.

Nevertheless, the econazole peak level observed after the fixed-dose combination application was far lower than that reported for the marketed 150 mg econazole ovules (7.5 ± 4.2 ng/mL vs. 65 ng/mL) [24]. Also, the highest mean benzydamine concentration at Day 3 was 11.0 ± 4.7 ng/mL, consistent with the peak concentration reported in the literature for vaginal benzydamine formulations (10 ± 8 ng/mL, 18 ng/mL, and 11.0 ± 2.7 ng/mL according, respectively, to [29–31]).

The present study results confirm that econazole and benzydamine are generally not absorbed from the fixed-dose combination through skin or mucosae to a significant amount, which limits the systemic exposure to the compounds and consequently the occurrence of untoward systemic effects. The vaginal route of administration may optimise the local action of the drugs by delivering them directly to the inflammation site.

A Phase III clinical trial demonstrated the antimicrobial effect and clinical efficacy of the fixed-dose combination in comparison to the marketed 150 mg econazole ovules, administered for 3 days in the treatment of uncomplicated vulvovaginal candidosis [40].

In conclusion, the present study showed a low systemic absorption of the fixed-dose combination active ingredients, which exclude any potential accumulation effects and limit the risk of undesired systemic side effects, thus ensuring a very good safety and tolerability profile in agreement with the safety data on both well-known active substances available in the literature.

## Figures and Tables

**Table 1:** Mean (±SD) baseline demographic data (*N* = 49).

Age (y)	Height (cm)	BW (kg)	BMI (kg/m^2^)	Race
38.2 ± 10.1	164.0 ± 5.2	63.44 ± 9.54	23.54 ± 2.86	White 45 (91.8%) Black 2 (4.1%) Mestizo 2 (4.1%)

BW: body weight; BMI: body mass index.

**Table 2:** Mean (±SD) AUC_0‐*t*_ (ng/mL × h) of plasma econazole measured and calculated on Days 1 and 3 of treatment with T and R1 and of plasma benzydamine and benzydamine N-oxide measured and calculated on Days 1 and 3 of treatment with T and R2.

Analyte	Day 1		Day 3	
	T *N* = 10	R1 *N* = 6	T *N* = 12	R1 *N* = 12
Econazole	89.710 ± 57.988	53.040 ± 40.870	149.645 ± 65.397	81.773 ± 51.338
	T *N* = 13	R2 *N* = 12	T *N* = 13	R2 *N* = 12
Benzydamine	100.965 ± 54.342	60.996 ± 34.305	220.214 ± 95.712	100.264 ± 52.733
Benzydamine N-oxide	26.562 ± 13.714	17.284 ± 12.051	69.877 ± 27.477	30.477 ± 14.612

AUC_0‐*t*_: area under the concentration curve from administration to the last observed concentration time *t*; T: test fixed-dose of econazole nitrate 150 mg/benzydamine hydrochloride 6 mg vaginal ovules; R1: reference 1 econazole nitrate 150 mg only ovules; R2: reference 2 benzydamine hydrochloride 6 mg only ovules.

**Table 3:** Number of subjects reporting and number of reported TEAEs by treatment, system organ class (SOC), and preferred term (PT) (safety set) *N* = 49.

MedDRA description SOC and PT term	T *N* = 13		R1 *N* = 12		R2 *N* = 12		P *N* = 12	
	AEs *n*	Subjects *n* (%)	AEs *n*	Subjects *n* (%)	AEs *n*	Subjects *n* (%)	AEs *n*	Subjects *n* (%)
*Total number of AEs and of subjects with at least one AE*	7	7 (53.8)	9	5 (41.7)	2	2 (16.7)	1	1 (8.3)
*General disorders and administration site conditions*	3	3 (23.1)	5	4 (33.3)	0	0	0	0
Application site reaction	0	0	4	4 (33.3)	0	0	0	0
Application site pruritus	2	2 (15.4)	1	1 (8.3)	0	0	0	0
Application site pain	1	1 (7.7)	0	0	0	0	0	0
*Investigations*	0	0	2	2 (16.7)	2	2 (16.7)	1	1 (8.3)
Gardnerella test positive	0	0	1	1 (8.3)	1	1 (8.3)	0	0
Candida test positive	0	0	0	0	1	1 (8.3)	0	0
Chlamydia test positive	0	0	0	0	0	0	1	1 (8.3)
Streptococcus test positive	0	0	1	1 (8.3)	0	0	0	0
*Nervous system disorders*	4	4 (30.8)	1	1 (8.3)	0	0	0	0
Headache	3	3 (23.1)	1	1 (8.3)	0	0	0	0
Dizziness	1	1 (7.7)	0	0	0	0	0	0
*Gastrointestinal disorders*	0	0	1	1 (8.3)	0	0	0	0
Nausea	0	0	1	1 (8.3)	0	0	0	0

TEAE: treatment emergent adverse event; AE: adverse event; T: test fixed-dose combination of econazole nitrate 150 mg/benzydamine hydrochloride 6 mg vaginal ovules; R1: reference 1 econazole nitrate 150 mg only ovules; R2: reference 2 benzydamine hydrochloride 6 mg only ovules; P: placebo vaginal ovules. MedDRA version 19.0.

## Data Availability

The primary study variable data used to support the findings of this study are completely included within this article.
